# Thresholds of polarization vision in octopuses

**DOI:** 10.1242/jeb.240812

**Published:** 2021-04-15

**Authors:** Shelby E. Temple, Martin J. How, Samuel B. Powell, Viktor Gruev, N. Justin Marshall, Nicholas W. Roberts

**Affiliations:** 1 Ecology of Vision Group, School of Biological Sciences, University of Bristol, Bristol BS8 1TQ, UK; 2 Azul Optics Ltd, Henleaze, Bristol BS9 4QG, UK; 3 Sensory Neurobiology Group, Queensland Brain Institute, University of Queensland, Brisbane, QLD 4072, Australia; 4 Biosensors Lab, Electrical and Computer Engineering, University of Illinois, Urbana, IL 61801, USA

**Keywords:** Degree of linear polarization, Cephalopod, Sensory ecology, Looming, Just-noticeable-difference

## Abstract

Polarization vision is widespread in nature, mainly among invertebrates, and is used for a range of tasks including navigation, habitat localization and communication. In marine environments, some species such as those from the Crustacea and Cephalopoda that are principally monochromatic, have evolved to use this adaptation to discriminate objects across the whole visual field, an ability similar to our own use of colour vision. The performance of these polarization vision systems varies, and the few cephalopod species tested so far have notably acute thresholds of discrimination. However, most studies to date have used artificial sources of polarized light that produce levels of polarization much higher than found in nature. In this study, the ability of octopuses to detect polarization contrasts varying in angle of polarization (AoP) was investigated over a range of different degrees of linear polarization (DoLP) to better judge their visual ability in more ecologically relevant conditions. The ‘just-noticeable-differences’ (JND) of AoP contrasts varied consistently with DoLP. These JND thresholds could be largely explained by their ‘polarization distance’, a neurophysical model that effectively calculates the level of activity in opposing horizontally and vertically oriented polarization channels in the cephalopod visual system. Imaging polarimetry from the animals’ natural environment was then used to illustrate the functional advantage that these polarization thresholds may confer in behaviourally relevant contexts.

## Abstract (precis)

**Summary:** Octopuses are highly sensitive to small changes in the angle of polarization (<1 deg contrast), even when the degree of polarization is low, which may confer a functional advantage in behaviourally relevant contexts.

## INTRODUCTION

Polarization vision is widespread in nature (Roberts et al., 2011), mainly among the invertebrates, where it contributes to a variety of behavioural tasks including navigation (Wehner, 1976), habitat localization (Schwind, 1991) and communication (Chiou et al., 2008). In terrestrial environments, this sensory capacity is best understood in the dorsal rim area of the eye, which is directed towards the sky to enable the detection of the pattern of celestial polarization (Wehner, 1976). Whole-field (whole-eye) polarization sensitivity also exists in many insects, for example butterflies (Kelber et al., 2001), moths (Belušič et al., 2017), dragonflies (Laughlin, 1976) and biting flies (Meglič et al., 2019), but the functional significance is less well studied (Heinloth et al., 2018; Kelber et al., 2001). Underwater, polarization sensitivity can also be found across the whole eye of many animal species, such as all cephalopods and most crustaceans (Fineran and Nicol, 1978; Moody and Parriss, 1961; Talbot and Marshall, 2011; Waterman et al., 1969). With polarization sensitivity across the whole visual field, polarization information can be used for diverse functions such as prey or predator detection (Temple et al., 2012) and communication (Cronin et al., 2003; Marshall et al., 2019; Marshall et al., 2014). This is partly because many aquatic environments are surrounded by stable polarization backgrounds (Cronin and Shashar, 2001; but see Johnsen et al., 2011; Shashar et al., 2000; Shashar et al., 1998). Additionally, several species of crustacean and cephalopod incorporate optical structures in their cuticle or skin, which produce strongly polarized signals for communication (Chiou et al., 2008; Marshall et al., 2019; Shashar et al., 1996).

It has been proposed that, in some underwater situations, polarization vision may prove more reliable than colour vision (Marshall and Cronin, 2011). Light transmission through seawater is strongly wavelength dependent, with short and long wavelengths being attenuated more rapidly than medium wavelengths (∼475 nm) (Smith and Baker, 1981). As a result, colour vision becomes less useful with depth (Bowmaker, 1995; Lythgoe, 1979; McFarland and Munz, 1975) and animals tend to tune their colour vision systems to take advantage of the wavelengths of light available to them (Cheroske and Cronin, 2003; Cheroske and Cronin, 2005; Cummings and Partridge, 2001; Lythgoe, 1968). The full range of polarization contrasts, however, can be present at any depth, so perhaps for this reason many marine animals may rely more heavily on polarization vision than colour. It must be noted though, that polarization contrasts are quickly eroded by veiling light and so are only effective over relatively short distances underwater (<12 m; Johnsen et al., 2016).

Cephalopods are a prime example of polarization specialists. Their advanced camera-type eyes use only a single type of visual pigment, rendering their visual world entirely monochromatic (Chung and Marshall, 2016; Marshall and Messenger, 1996; Mathger et al., 2006; Messenger, 1977). Instead, their rhabdomeric photoreceptors are precisely ordered into two channels of polarization sensitivity, resulting in dipolatic vision across the whole visual field (Labhart, 2016; Moody and Parriss, 1961; Talbot and Marshall, 2011). This provides a highly sensitive polarization vision system ideally suited to detecting object-based contrasts in their marine environment (Shashar and Cronin, 1996; Temple et al., 2012), roughly analogous to how we use our own colour vision system. Previous studies with cephalopods have demonstrated both neural and behavioural responses to very small contrasts in polarization (Saidel et al., 2005; Temple et al., 2012). However, these studies used stimuli with degrees of polarization much higher (∼1.0) than those found in nature (<0.7) (Horváth et al., 2015; Novales Flamarique and Hawryshyn, 1997) and so do not represent the range of contrasts typically encountered by these animals in the wild. To investigate this further, we examined the threshold of detection of polarization contrasts in two species of octopus (*Abdopus aculeatus* and *Octopus cyanea*) by varying both angle of polarization (AoP) and degree of linear polarization (DoLP) using a modified liquid crystal display (LCD) to deliver dynamic polarization stimuli (Basnak et al., 2018; Glantz and Schroeter, 2007; How et al., 2012; Pignatelli et al., 2011; Temple et al., 2015). We compared their polarization sensitivity with measurements of polarization taken from their natural environment.

## MATERIALS AND METHODS

Experiments were performed during four visits to the Lizard Island Research Station, Australia (location: 14°40′03″S; 145°26′49″E) between 2012 and 2015. Ten octopuses [8 *Abdopus aculeatus* (d'Orbigny 1834) and 2 *Octopus cyanea* Gray 1849] were collected opportunistically at low tide on coral reefs around Lizard Island. Only individuals with a mantle length of approximately 7 cm or less were retained for testing; most were closer to 3–4 cm mantle length. Direct length and mass measurements were not made, to reduce stress to the animals. Octopuses were kept individually in glass aquaria (15×15×15 cm) continually supplied with flow-through filtered and oxygenated ocean water and maintained under a natural day–night light cycle. Glass lids were placed over the aquaria, weighted down with lead diver's weights to prevent escapes. Fresh crabs and stomatopods were fed to the animals daily, with the size/number of food items matched to the animal's size; if the animal fed eagerly, more was provided if it was available that day. Octopuses were released back to the immediate vicinity of their capture location after testing (<14 days).

Visual stimuli were presented to each individual octopus by placing the octopus, in its home aquarium, in front of a modified LCD computer monitor (15 inch LCD, Type: VPC15AS1, Viglen, St Albans, Hertfordshire, UK). By moving the animal in its home tank, we avoided the undue stress of repeatedly capturing the animals each time they were tested. The animal was given time to acclimatize to the new visual surroundings after being moved. Stimulus presentations commenced when the animal was stationary yet awake and at least one eye was looking at the stimulus screen; the time taken for this to occur varied greatly between animals. The aquarium containing the octopus could be rotated so that the octopus had a clear view of the screen and was in the back third of the tank, 10–16 cm from the screen. During testing, the bottom and the two sides of the tank perpendicular to the viewing surface of the LCD were lined with white felt to reduce internal reflections that create intensity artefacts (Foster et al., 2018). The LCD and aquarium were covered with a black-out cloth to avoid movements in the testing room disturbing the subjects and therefore the testing tank was illuminated by the light emitted by the LCD screen and a small amount of room light that leaked through and around the black-out cloth; the precise intensity of this background light was neither controlled nor measured and therefore may have added some variance to the threshold values recorded.

**Fig. 1.**
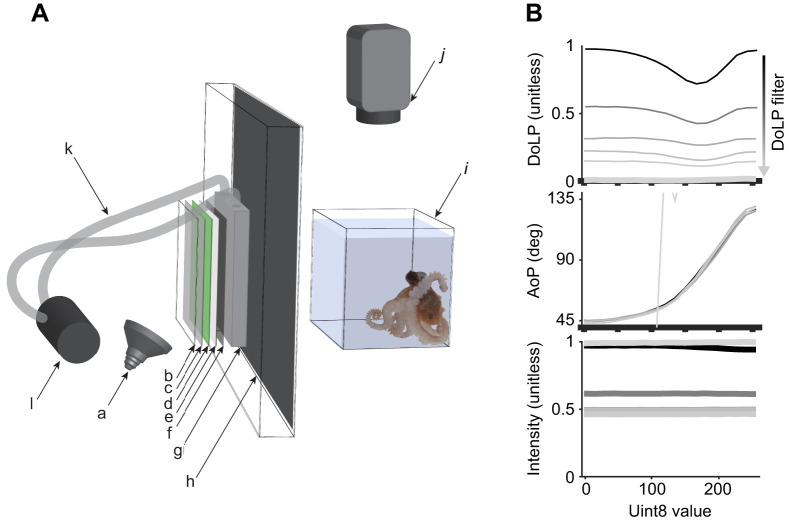
**Experimental setup and stimulus LCD measurements.** (A) Experimental apparatus (not to scale) used to present dynamic visual stimuli varying in the degree of linear polarization (DoLP) and angle of polarization (AoP). White light from an LED bulb (a) was projected off-axis through a series of filters that included: a clear sheet of acrylic (b); a neutral density filter (c); a green gelatin filter (d); a thin sheet of Teflon (e); and a polarizer (f). These have all been spaced apart in the diagram for the purposes of labelling but were held tightly against a small acrylic tank (g) filled with water and hollow glass spheres. The light was then transmitted through a modified LCD computer monitor (h) with its own light source removed along with both the front and back polarizers. The moving images created with this system were displayed to an octopus resting in a tank (i) adjacent to the monitor. The behavioural responses of the octopus were recorded with a video camera (j) mounted above. The AoP of the images presented to the octopus was controlled by the LCD, while the DoLP was controlled by varying the concentration of hollow glass spheres (scattering agent) in the acrylic tank (g). To maintain an even distribution of hollow glass spheres, a small pump (l), connected to the tank by tubes (k), maintained constant movement of the water in the acrylic tank. (B) Polarization and relative intensity characteristics of the stimulus LCD monitor at six of the nine different DoLP settings used, measured using a Glan–Thompson Fresnel Rhomb assembly coupled to a spectrophotometer. Uint8 value is an 8 bit LCD scale. Note that the AoP estimates at the lowest DoLP setting are unreliable, resulting in the steep diagonal light grey line in the AoP graph. See Fig. S2 for an expanded version.

The LCD was modified to display video in polarization contrast only, as described in Temple et al. (2012), and further modified so that the DoLP could be varied. Briefly, the front polarizer was removed such that the images varied in AoP rather than intensity. To control the DoLP, the LCD was further modified by removing the light supply and rear polarizer and replacing them with an LED light source (6 W, 4000 K, Master LED spot; MV GU10, Philips, Eindhoven, The Netherlands) that projected (off-axis at an angle of 30 deg) onto one of a series of custom designed DoLP filters positioned against the back side of the LCD. The DoLP filters included: (1) a thin sheet of acrylic; (2) a neutral density filter (varied ND filters, Lee Filters, Andover, Hampshire, UK); (3) a green gelatin filter (fern green #122, Lee Filters); (4) a thin (0.28 mm) sheet of Teflon to completely depolarize and spatially homogenize the light; (5) a sheet of polarizer (#7300, Rosco, London, UK); and (6) a diffusing/scattering tank to reduce the DoLP before it entered the liquid crystal matrix (Fig. 1A). The DoLP was varied by using different densities of hollow glass spheres (10 µm; Dantec Dynamics, Skovlunde, Denmark) suspended in water in the diffusing tanks (20×20×1 cm tanks constructed of 6 mm acrylic). A homogeneous distribution of the hollow glass spheres was maintained with vigorous flow provided by a 12 V water pump (automobile windshield washer fluid pump). Varying the DoLP resulted in changes in the overall intensity transmitted through the filters: the average change was 7%, with the total change in intensity from highest to lowest DoLP filter being 51% (Fig. 1B). To compensate for this, the intensity among the different DoLP filters was roughly matched by the addition of neutral density gelatin filters (item 2 in the list above) on the back (LED light) side of the Teflon sheet. The absolute spectral radiance of the LCD monitor was measured (Fig. S1) using a calibrated spectrophotometer (USB65000, Ocean Optics, Largo, FL, USA). The AoP of light emitted by the modified LCD varied from 45 to 130 deg in relation to the Uint8 value (8 bit LCD scale) that can be varied from 0 to 255. The background upon which the stimulus was shown was oriented horizontally (0 deg) by rotating the LCD by 45 deg. The depolarizing filters permitted the entire image to vary in DoLP (from 0.0 to 1.0) without altering the AoP contrast of the images displayed (Fig. 1B). The precise polarization characteristics of the monitor were measured using a Glan–Thompson Fresnel Rhomb assembly, coupled to a spectrophotometer (USB2000, Ocean Optics). Full methods for LCD measurement are published in Foster et al. (2018).

As reported previously (Foster et al., 2018; How et al., 2012; Temple et al., 2012), small changes in radiant energy are produced by our modified LCDs when viewed at oblique angles (away from normal to the plane of the screen). To minimize these effects, the looming image was centred on the animal's eyes for each presentation by adjusting the vertical and horizontal position of the centre of the looming object in the PowerPoint presentation in relation to the animal's position in the aquarium. This ensured that the largest angle subtended by the edge of the image was less than 10 deg from normal to the LCD surface. Additionally, small changes in DoLP accompanied the different AoP settings because of the presence of a variable component of ellipticity (Stokes vector S3) in the transmitted light from the display (see Foster et al., 2018, for details). These were measured and incorporated in the final analysis.

Octopuses were shown videos of a looming object (rapidly expanding circle) in polarization-only contrast. Looming stimuli were created using the ‘zoom in’ animation in PowerPoint (Microsoft Corporation, Albuquerque, NM, USA) (duration of expansion: 200 ms) and the stimulus disappeared 5.0 s later using the ‘shrink’ animation. The appearance and disappearance of the stimulus was marked by a click sound that was only audible on the video camera audio track and to the tester via a set of headphones. The stimulus was 4.0 cm onscreen and subtended an angle of less than 20 deg when viewed by the octopus. To determine an animal’s threshold, the AoP of the stimulus relative to the background was decreased until the animal stopped responding; the AoP was then decreased a further 1–2 steps to ensure that the animal could not detect the stimulus. Step sizes were 15 on the Uint8 scale (range of 0–255). This step size was decreased to steps of 5 Uint8 values when threshold values approached the limit of the monitor, e.g. background value of Uint8=0. When the threshold AoP was determined at one DoLP level, the same descending process through AoP values was repeated at the next lowest DoLP. At any DoLP value being tested, the lowest AoP at which a response was detected concomitant with the stimulus was determined as the animal's threshold (see below for a description of positive responses). Multiple passes of the descent through AoP values (repeats) were not completed because of the high number of different stimuli that needed to be presented (several AoP values at each of 9 DoLP settings), which would have put undue stress on the subjects. To maintain the subject's attention on the screen, periodically, higher contrast AoP stimuli were interjected into the descending pass. This enabled the observer to verify that the octopus was attentive to the stimuli (i.e. that the animal's eyes were open and that it was awake and looking in the direction of the stimulus, usually with one eye).

At the time of testing, the experimenter was able to observe the octopus’ responses on closed-circuit video capture and then replay the video recordings to determine whether a response had occurred. Responses took the form of changes in body colour pattern or movement of arms or papillae that followed the stimulus by precisely 200 ms and observably differed from any background variations in body pattern or movement. The presence of a response was evident to the trained observer; however, detection of very weak responses often required watching the video footage several times to confirm the response. If at the time of testing there was any doubt about whether the individual had responded or not, or if a background body pattern or movement change may have coincidently occurred simultaneously with the stimulus presentation, then that stimulus step was repeated, as the next stimulus or after a repeat of the previous few higher contrast stimuli. For scoring, all videos were analysed ‘blind’ by a trained observer at the end of the study without knowledge of stimulus setting presented. Subject responses to looming stimuli were categorized into one of five subjective response strength categories (0=no response, 1=very weak/just perceptible response, 2=weak response, 3=medium response, 4=strong response). Examples of responses for each category have been included in Movies 1 and 2 (also available from https://doi.org/10.5523/bris.1r4kwj2eu0tnq1yj9b9pdeb5bg). Stimuli were only presented to the octopus if it was awake, and had at least one eye open facing the screen, which was ascertained from the closed-circuit video feed. Data analysis was performed graphically with calculation of median, using Microsoft Excel and IBM SPSS V24.

Looming stimuli were presented every 2–5 min, a frequency that was found to allow octopuses to maintain strong responses for several hours over multiple days with no ill effects (i.e. the animals continued to be interactive during regular husbandry interactions and fed well throughout their stay in the laboratory). The threshold for angular contrast was determined at nine settings of DoLP (0.005, 0.04, 0.08, 0.15, 0.22, 0.31, 0.55, 0.75, 0.98). Note that not all individuals contributed data for each DoLP setting, because of experimental limitations.

Our measure of ‘polarization distance’ has been presented in detail in How and Marshall (2014). The model is based on the principle outlined by Bernard and Wehner (1977), that polarization-sensitive photoreceptors react to stimulus light sources according to the following formula:(1)
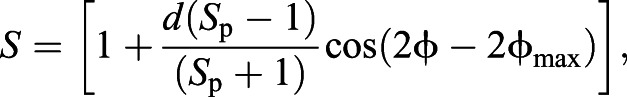
where *S* is receptor sensitivity to incoming light, *S*_p_ denotes overall sensitivity calculated as the maximum divided by minimum response potential, *d* is the degree of polarization (DoP), φ is the angle of polarization (AoP) and φ_max_ is the AoP to which the photoreceptor cell is maximally sensitive.

The signal from these two photoreceptors is then compared in a hypothetical interneuron using the following formula:(2)
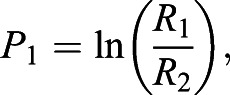
where *P*_1_ is the response of the first opponent interneuron, and *R*_1_ and *R*_2_ are the two polarization-sensitive photoreceptors oriented perpendicularly to each other.

To calculate a measure of contrast between receptors looking at an object compared with receptors looking at the background, the responses of *P*_1_ interneurons pointing at these two points in space are compared by a second hypothetical interneuron to produce a value of polarization distance as follows:(3)
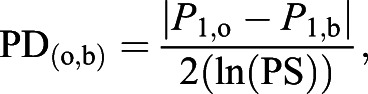
where PD is the polarization distance, o is the object, b is the background and PS is the polarization sensitivity of the receptor (in the case of this study, a precise PS value is unknown for cephalopods, so we used an assumed high PS of 10).

The script used for calculating PD is included in the supplementary information (Script 1). Written in Matlab (Natick, MA, USA), Script 1 implements Bernard and Wehner's (1977) receptor sensitivity equations and How and Marshall's (2014) polarization distance calculations. Script 1 is fully annotated and provides a step-by-step approach to the method.

## RESULTS

**Fig. 2.**
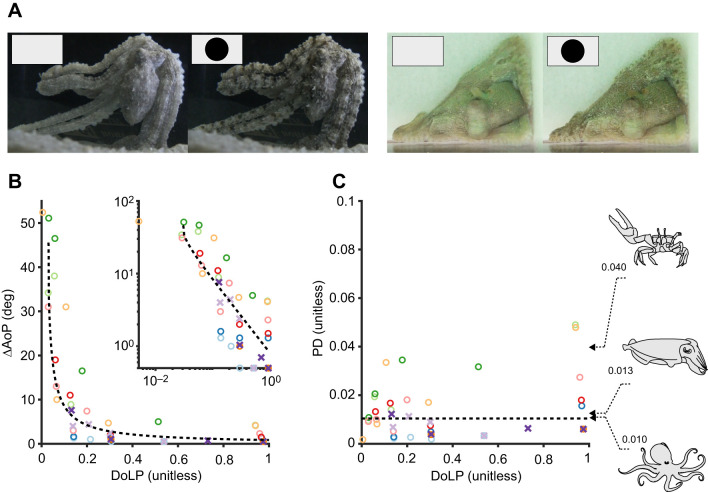
**Response thresholds of octopus to looming stimuli varying in DoLP and ΔAoP.** (A) Example responses from two different individual octopuses to looming stimuli. The black looming stimulus in the inset is for illustration – the animal experienced the stimulus in polarization contrast only. (B) Linear and logarithmic (inset) plots of the threshold response for each individual (*n*=10; colour coded; circles, *Abdopus aculeatus*; crosses, *Octopus cyanea*). The dotted line indicates the projection of median threshold of polarization distance (PD; from C). (C) The same response threshold data plotted as a function of PD (see How and Marshall, 2014, for calculation). The black dotted line indicates the median PD. Results from previous investigations of cuttlefish (Temple et al., 2012) and fiddler crabs (How et al., 2012) are given for comparison.

Octopuses responded to looming stimuli typically within milliseconds, with changes in body colour pattern (Fig. 2A). These changes in body pattern were approximately proportional to the contrast of the stimulus relative to the background, as observed previously in cuttlefish (Temple et al., 2012), such that high contrast stimuli invoked a full body colour change often combined with movement, while low contrast stimuli invoked small changes in body colour often restricted to one part of the body, e.g. a few square millimetres of one arm (see Movies 1 and 2, also available at https://doi.org/10.5523/bris.1r4kwj2eu0tnq1yj9b9pdeb5bg). We saw no difference (quantitative or qualitative) in the responses of the two species, and the threshold values of the two *O. cyanea* individuals fell within the range measured for the eight *A. aculeatus*.

When the DoLP was high (>0.3) the animals were able to respond to differences in the AoP of the stimulus relative to the background (ΔAoP) at a median value of 1.3 deg. Below a DoLP of 0.3, the minimum angular contrast between stimulus and background required to elicit a response increased rapidly as DoLP approached 0 (Fig. 2B).

An alternative system for representing polarization contrasts is to use a measure of ‘polarization distance’ or PD. Roughly analogous to the better-known ‘colour distance’ measure, PD provides an estimate of the amount of contrast detectable to a given polarization vision system (in this case, a dipolat with horizontal and vertical polarization receptors). For a full explanation of PD, see How and Marshall (2014); the equations and Matlab script used to calculate PD in this study are included in Script 1. When plotted as a function of PD, the measured thresholds line up approximately along a median PD value of 0.010 (Fig. 2C). The relationship between this threshold value and the range of DoLP and ΔAoP stimuli is illustrated by projecting the threshold onto the initial stimulus axes (Fig. 2B, dotted black line). This demonstrates that much of the variance in the data can be explained by the orientation of the underlying polarization-sensitive photoreceptors on which the polarization distance model is based. Some of the remaining variance can be explained by performance differences between individuals, with mean PD threshold across all ΔAoP/DoLP combinations ranging from 0.0049 to 0.024.

## DISCUSSION

Octopuses responded to looming stimuli varying in polarization contrast alone and were sensitive to very small ΔAoP, particularly when the DoLP was high. The large variation in ΔAoP required to elicit a response (1 deg at high DoLP, to 53 deg for low DoLP) may be accounted for using the ‘polarization distance’ neurophysical model first suggested by Bernard and Wehner (1977) and then later expanded upon by How and Marshall (2014). This model takes a neural-processing approach to understanding contrast from the perspective of the animal's polarization vision system. Because cephalopods use a dipolatic system based on two polarization channels oriented horizontally and vertically relative to the outside world (Labhart, 2016; Moody and Parriss, 1961; Talbot and Marshall, 2011), AoP contrasts at low DoLP need to be larger to elicit an equivalent contrast in the photoreceptor output compared with stimuli at high DoLP. Along with the work of Basnak et al. (2018), this study is among the first to show how the systematic probing of polarization contrast sensitivity across the range of DoLP can converge on a single value of PD, reinforcing the validity of this approach to the study of polarization-based contrast vision in animals.

The sensitivity of polarization-based contrast vision has been measured previously for several other animals (see below), but the median behavioural threshold of PD=0.010 recorded in this study is the most acute recorded so far. Furthermore, several individuals responded to stimuli with PD values well below 0.010 (Fig. 2C). For example, the 10 most acute thresholds of response measured in the study ranged between PD=0.0017 and 0.0039. That responses were not always detected below PD=0.010 may reflect variance in motivation state, habituation or stress of the animals at the time of testing, which is difficult to control, or the lack of sensitivity of our response detection system, which relied on observing small changes in colour pattern only. As such, these results could be considered as conservative, as we suspect that the actual behavioural threshold may be closer to PD=0.002–0.004, but confirmation of this will require further investigation.

In other animals, the performance of the dorsal rim area of insects has been investigated in crickets and bees, which show electrophysiological and behavioural responses to dorsally presented polarization patterns down to a DoLP of 0.05–0.10 (Henze and Labhart, 2007; Labhart, 1996; von Frisch, 1967). This kind of celestial polarization vision operates using different requirements to object-based vision, in that the aim is to encode information about the overall AoP of the wide-field sky pattern rather than detecting contrasts between objects and background in an image parsing approach. As such, it is not meaningful to compare these thresholds directly with those measured in the current study. In crustaceans, the dorsal light reflex of crayfish can be reliably elicited by polarization contrasts as low as a ΔAoP of 15.2 deg (at DoLP of 1.0) and a DoLP of 0.13 (at ΔAoP of 20 deg) (Glantz and Schroeter, 2006). The conversion of these values to PD is not possible because of a lack of information about absolute AoP within the stimuli, but as an approximate comparison, the octopuses responded to a median ΔAoP of 1.3 deg when the DoLP was >0.3, an order of magnitude more acute than the equivalent threshold in the crayfish. In other studies, the startle behaviour of fiddler crabs was measured to a threshold ΔAoP of 3.2 deg and DoLP of 0.08, equivalent to PD=0.040 and 0.075, respectively (How et al., 2014; How et al., 2012). Stomatopods performed less well, only responding to contrasts of DoLP that are greater than ∼0.2 (when AoP for stimulus and background is 90 deg) (How et al., 2014), equivalent to PD=0.16, although the behaviours tested may not have revealed the absolute limit of PD threshold in these species. The cuttlefish *Sepia plangon* has also been tested using a very similar approach to the current study and reached a threshold performance of ΔAoP of 1.05 deg at a DoLP of 1.0 (PD=0.013) (Temple et al., 2012). The median value of PD=0.010 reported here is similar to that reported in *S. plangon*, but the frequency of responses at thresholds as low as PD=0.002 suggests that technological and methodological improvements in testing these and other cephalopods may well be rewarded with a more accurate estimate of their true abilities.

**Fig. 3.**
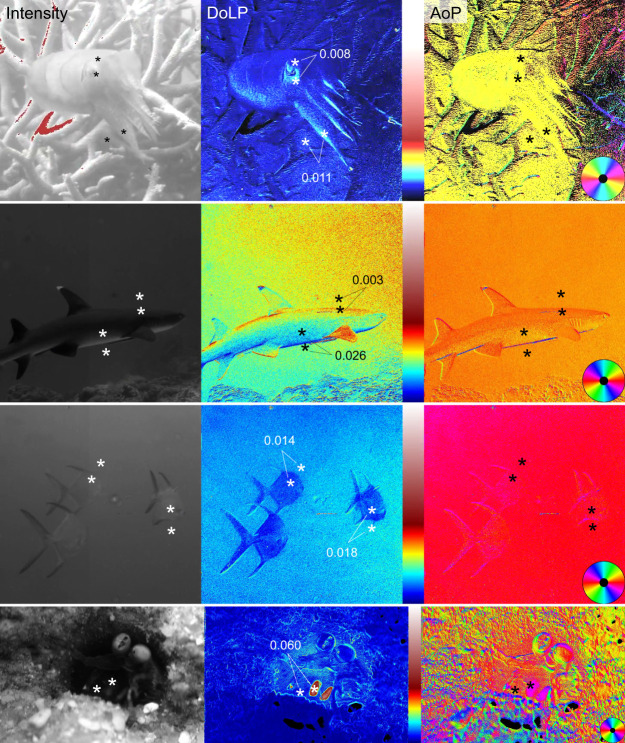
**Underwater polarimetry from the Great Barrier Reef, Australia.** Each panel consists of an intensity (left), DoLP (middle) and AoP (right) image. DoLP and AoP panels are false-coloured according to the scale in each image. For DoLP the scale ranges from 0 to 1. See Johnsen et al. (2016) and York et al. (2014) for detailed methods. Top row: a cuttlefish among coral. Second row: a shark against an open water background. Third row: silvery fishes against an open water background. Bottom row: a stomatopod in the entrance to its burrow with two large oval eyes (top right) and below them towards the centre of the image its highly polarized appendages, which are barely visible in the intensity channel but show high contrast in the DoLP channel. Representative examples of PD for pairs of locations in the image are illustrated in the central panels. Note that the PD values use both DoLP and AoP measurements. All but two of the PDs labelled in these images would be visible to an octopus based on the median value reported here of PD=0.010; however, the lower limit of measurements in half of the animals tested suggests that all of these PDs would be detectable if the threshold were as low as the possible PD=0.002.

Assessing the functional advantage of the sensitive polarization vision in colourblind octopuses requires investigation of the types of visual scenes these animals may experience. Photographic polarimetry from the natural environment shows a range of polarization contrasts that fall close to or within the range of detection of the octopus visual system (Fig. 3) (Johnsen et al., 2016; Marshall et al., 2019). Cues and signals from fish predators and prey, as well as the communication signals of cephalopods and other animals, all generate polarization contrasts well within the detection range measured in this study.

Polarization contrast may add another channel of visual information that can be used in conjunction with intensity for parsing objects from the polarized backdrop of the underwater light field, or for identifying hitherto undiscovered communication signals from conspecifics. Whether polarization contrast acts as an independent channel of information to the intensity variations that frequently co-occur, or whether it simply modulates intensity to enhance a single contrast channel (analogous to wearing polaroid sunglasses) remains to be demonstrated. Inspiration for how to approach this question could come from recent work on fiddler crabs *Afruca tangeri* (Smithers et al., 2019), which made use of spatiotemporally synchronized intensity and polarization LCD displays to show that they respond to intensity and polarization contrasts as separate channels of information.

While our results point to a remarkably sensitive polarization contrast detection system in octopuses, it is worth mentioning two things: firstly, it is unlikely to be unique to the specific species studied here; and secondly, we know very little about how octopuses might use polarization sensitivity in their everyday behaviours. Our preliminary results with other cephalopods (cuttlefish and squid) point to equal sensitivity in other species and these are known to specifically target silvery fish against open waters where polarization vision may help (Shashar et al., 2000). We hope that our experimental paradigm combined with other new approaches like 3D glasses mounted on cuttlefish (Feord et al., 2020) and more behaviourally relevant stimuli may lead to a comprehensive understanding of this exciting alternative to colour vision.

## Supplementary Material

Supplementary information

## References

[JEB240812C1] Basnak, M. A., Pérez-Schuster, V., Hermitte, G. and Berón de Astrada, M. (2018). Polarized object detection in crabs: a two-channel system. *J. Exp. Biol.* 221, jeb173369. 10.1242/jeb.173369 29650753

[JEB240812C2] Belušič, G., Šporar, K. and Meglič, A. (2017). Extreme polarisation sensitivity in the retina of the corn borer moth *Ostrinia*. *J. Exp. Biol.* 220, 2047. 10.1242/jeb.153718 28341662

[JEB240812C3] Bernard, G. D. and Wehner, R. (1977). Functional similarities between polarization vision and color vision. *Vision Res.* 17, 1019-1028. 10.1016/0042-6989(77)90005-0 595410

[JEB240812C4] Bowmaker, J. K. (1995). The visual pigments of fish. *Prog. Retin. Eye Res.* 15, 1-27. 10.1016/1350-9462(95)00001-1

[JEB240812C5] Cheroske, A. G. and Cronin, T. W. (2003). Phenoptypically plastic color vision in a stomatopod crustacean and its potential effects on color signaling in variable light environments. *Integrative and Comparative Biology* 43, 1015-1015.

[JEB240812C6] Cheroske, A. G. and Cronin, T. W. (2005). Variation in stomatopod (Gonodactylus smithii) color signal design associated with organismal condition and depth. *Brain Behav. Evol.* 66, 99-113. 10.1159/000086229 15942161

[JEB240812C7] Chiou, T. H., Caldwell, R. L., Hanlon, R. T. and Cronin, T. W. (2008). Fine structure and optical properties of biological polarizers in crustaceans and cephalopods. In *Polarization: Measurement, Analysis, and Remote Sensing VIII*, vol. 6972 (ed. D. B. Chenault and D. H. Goldstein) , p. 697203. SPIE.

[JEB240812C8] Chung, W.-S. and Marshall, N. J. (2016). Comparative visual ecology of cephalopods from different habitats. *Proceedings of the Royal Society B* 283, 20161346. 10.1098/rspb.2016.1346 27629028 PMC5031660

[JEB240812C9] Cronin, T. W. and Shashar, N. (2001). The linearly polarized light field in clear, tropical marine waters: Spatial and temporal variation of light intensity, degree of polarization and e-vector angle. *J. Exp. Biol.* 204, 2461-2467. 11511661 10.1242/jeb.204.14.2461

[JEB240812C10] Cronin, T. W., Shashar, N., Caldwell, R. L., Marshall, J., Cheroske, A. G. and Chiou, T. H. (2003). Polarization signals in the marine environment. *Polarization Science and Remote Sensing* 5158, 85-92. 10.1117/12.507903

[JEB240812C11] Cummings, M. and Partridge, J. (2001). Visual pigments and optical habitats of surfperch (Embiotocidae) in the California kelp forest. *Journal of Comparative Physiology A* 187, 875-889. 10.1007/s00359-001-0258-6 11866186

[JEB240812C12] Feord, R. C., Sumner, M. E., Pusdekar, S., Kalra, L., Gonzalez-Bellido, P. T. and Wardill, T. J. (2020). Cuttlefish use stereopsis to strike at prey. *Sci. Adv.* 6, eaay6036. 10.1126/sciadv.aay6036 31934631 PMC6949036

[JEB240812C13] Fineran, B. A. and Nicol, J. A. C. (1978). Studies on the photoreceptors of *Anchoa mitchilli* and *A. hepsetus* (Engraulidae) with particular reference to the cones. *Phil. Trans. R. Soc. B* 283, 25-60. 10.1098/rstb.1978.0017 17130

[JEB240812C14] Foster, J. J., Temple, S. E., How, M. J., Daly, I. M., Sharkey, C. R., Wilby, D. and Roberts, N. W. (2018). Polarisation vision: overcoming challenges of working with a property of light we barely see. *Naturwissenschaften* 105, 27-27. 10.1007/s00114-018-1551-3 PMC5871655 29589169

[JEB240812C15] Glantz, R. and Schroeter, J. (2006). Polarization contrast and motion detection. *Journal of Comparative Physiology A: Neuroethology, Sensory, Neural, and Behavioral Physiology* 192, 905-914. 10.1007/s00359-006-0127-4 16830137

[JEB240812C16] Glantz, R. M. and Schroeter, J. P. (2007). Orientation by polarized light in the crayfish dorsal light reflex: behavioral and neurophysiological studies. *Journal of Comparative Physiology A-Neuroethology Sensory Neural and Behavioral Physiology* 193, 371-384. 10.1007/s00359-006-0191-9 17143624

[JEB240812C17] Heinloth, T., Uhlhorn, J. and Wernet, M. F. (2018). Insect responses to linearly polarized reflections: orphan behaviors without neural circuits. *Front. Cell. Neurosci.* 12. 10.3389/fncel.2018.00050 PMC5870057 29615868

[JEB240812C18] Henze, M. J. and Labhart, T. (2007). Haze, clouds and limited sky visibility: polarotactic orientation of crickets under difficult stimulus conditions. *J. Exp. Biol.* 210, 3266. 10.1242/jeb.007831 17766304

[JEB240812C19] Horváth, G., Barta, A. and Hegedüs, R. (2015). Polarization of the sky. In *Polarized light and polarization vision in animal sciences* (ed. G. Horváth), pp. 367-406. Springer.

[JEB240812C20] How, M. J. and Marshall, N. J. (2014). Polarization distance: a framework for modelling object detection by polarization vision systems. *Proceedings of the Royal Society B* 281, 20131632. 10.1098/rspb.2013.1632 24352940 PMC3871304

[JEB240812C21] How, M. J., Pignatelli, V., Temple, S. E., Marshall, N. J. and Hemmi, J. M. (2012). High e-vector acuity in the polarisation vision system of the fiddler crab *Uca vomeris*. *J. Exp. Biol.* 215, 2128-2134. 10.1242/jeb.068544 22623201

[JEB240812C22] How, M. J., Christy, J., Roberts, N. W. and Marshall, N. J. (2014). Null point of discrimination in crustacean polarisation vision. *J. Exp. Biol.* 217, 2462-2467. 10.1242/jeb.103457 24737768

[JEB240812C23] Johnsen, S., Marshall, N. J. and Widder, E. A. (2011). Polarization sensitivity as a contrast enhancer in pelagic predators: lessons from *in situ* polarization imaging of transparent zooplankton. *Phil. Trans. R. Soc. B* 366, 655-670. 10.1098/rstb.2010.0193 21282169 PMC3049004

[JEB240812C24] Johnsen, S., Gagnon, Y. L., Marshall, N. J., Cronin, T. W., Gruev, V. and Powell, S. (2016). Polarization vision seldom increases the sighting distance of silvery fish. *Curr. Biol.* 26, R752-R754. 10.1016/j.cub.2016.07.030 27554649

[JEB240812C25] Kelber, A., Thunell, C. and Arikawa, K. (2001). Polarisation-dependent colour vision in *Papilio* butterflies. *J. Exp. Biol.* 204, 2469-2480. 11511662 10.1242/jeb.204.14.2469

[JEB240812C26] Labhart, T. (1996). How polarization-sensitive interneurones of crickets perform at low degrees of polarization. *J. Exp. Biol.* 199, 1467. 9319364 10.1242/jeb.199.7.1467

[JEB240812C27] Labhart, T. (2016). Can invertebrates see the e-vector of polarization as a separate modality of light? *J. Exp. Biol.* 219, 3844-3856. 10.1242/jeb.139899 27974532 PMC5201003

[JEB240812C28] Laughlin, S. B. (1976). The sensitivities of dragonfly photoreceptors and the voltage gain of transduction. *J. Comp. Physiol.* 111, 221-247. 10.1007/BF00606466

[JEB240812C29] Lythgoe, J. N. (1968). Visual pigments and visual range underwater. *Vision Res.* 8, 997-1011. 10.1016/0042-6989(68)90073-4 5683090

[JEB240812C30] Lythgoe, J. N. (1979). *The ecology of vision*. Oxford: Clarendon Press.

[JEB240812C31] Marshall, J. and Cronin, T. W. (2011). Polarisation vision. *Curr. Biol.* 21, R101-R105. 10.1016/j.cub.2010.12.012 21300269

[JEB240812C32] Marshall, N. J. and Messenger, J. B. (1996). Colour-blind camouflage. *Nature* 382, 408-409. 10.1038/382408b08684479

[JEB240812C33] Marshall, N. J., Roberts, N. W. and Cronin, T. W. (2014). Polarisation signals. In *Polarized light and polarization vision in animal sciences* (ed. G. Horvath), pp. 407-442. New York: Springer.

[JEB240812C34] Marshall, N. J., Powell, S. B., Cronin, T. W., Caldwell, R. L., Johnsen, S., Gruev, V., Chiou, T.-H. S., Roberts, N. W. and How, M. J. (2019). Polarisation signals: a new currency for communication. *J. Exp. Biol.* 222, 16. 10.1242/jeb.134213 30733259

[JEB240812C35] Mäthger, L. M., Barbosa, A., Miner, S. and Hanlon, R. T. (2006). Color blindness and contrast perception in cuttlefish (*Sepia officinalis*) determined by a visual sensorimotor assay. *Vision Res.* 46, 1746-1753. 10.1016/j.visres.2005.09.035 16376404

[JEB240812C36] McFarland, W. N. and Munz, F. W. (1975). Part II: The photic environment of clear tropical seas during the day. *Vision Res.* 15, 1063-1070. 10.1016/0042-6989(75)90002-4 1166605

[JEB240812C37] Meglič, A., Ilić, M., Pirih, P., Škorjanc, A., Wehling, M. F., Kreft, M. and Belušič, G. (2019). Horsefly object-directed polarotaxis is mediated by a stochastically distributed ommatidial subtype in the ventral retina. *Proc. Natl Acad. Sci. USA* 116, 21843. 10.1073/pnas.1910807116 31591223 PMC6815168

[JEB240812C38] Messenger, J. B. (1977). Evidence that *Octopus* is colour blind. *J. Exp. Biol.* 70, 49.

[JEB240812C39] Moody, M. F. and Parriss, J. R. (1961). The discrimination of polarized light by Octopus: a behavioural and morphological study. *Zeitschrift für vergleichende Physiologie* 44, 268-291. 10.1007/BF00298356

[JEB240812C40] Novales Flamarique, I. and Hawryshyn, C. W. (1997). Is the use of underwater polarized light by fish restricted to crepuscular time periods? *Vision Res.* 37, 975-989. 10.1016/S0042-6989(96)00236-2 9196717

[JEB240812C41] Pignatelli, V., Temple, S. E., Chiou, T.-H., Roberts, N. W., Collin, S. P. and Marshall, N. J. (2011). Behavioural relevance of polarization sensitivity as a target detection mechanism in cephalopods and fishes. *Phil. Trans. R. Soc. B Biol. Sci.* 366, 734-741. 10.1098/rstb.2010.0204 PMC3049012 21282177

[JEB240812C42] Roberts, N. W., Porter, M. L. and Cronin, T. W. (2011). The molecular basis of mechanisms underlying polarization vision. *Philosophical Transactions of the Royal Society B: Biological Sciences* 366, 627-637. 10.1098/rstb.2010.0206 PMC3049014 21282166

[JEB240812C43] Saidel, W. M., Shashar, N., Schmolesky, M. T. and Hanlon, R. T. (2005). Discriminative responses of squid (*Loligo pealeii*) photoreceptors to polarized light. *Comparative Biochemistry and Physiology Part A: Molecular & Integrative Physiology* 142, 340-346. 10.1016/j.cbpa.2005.08.003 16165381

[JEB240812C44] Schwind, R. (1991). Polarization vision in water insects and insects living on a moist substrate. *Journal of Comparative Physiology A: Neuroethology, Sensory, Neural, and Behavioral Physiology* 169, 531-540. 10.1007/BF00193544

[JEB240812C45] Shashar, N. and Cronin, T. W. (1996). Polarization contrast vision in *Octopus*. *J. Exp. Biol.* 199, 999-1004. 8788092 10.1242/jeb.199.4.999

[JEB240812C46] Shashar, N., Rutledge, P. S. and Cronin, T. W. (1996). Polarization vision in cuttlefish - A concealed communication channel? *J. Exp. Biol.* 199, 2077-2084. 9319987 10.1242/jeb.199.9.2077

[JEB240812C47] Shashar, N., Hanlon, R. T. and Petz, A. D. (1998). Polarization vision helps detect transparent prey. *Nature* 393, 222-223. 10.1038/303809607759

[JEB240812C48] Shashar, N., Hagan, R., Boal, J. G. and Hanlon, R. T. (2000). Cuttlefish use polarization sensitivity in predation on silvery fish. *Vision Res.* 40, 71-75. 10.1016/S0042-6989(99)00158-3 10768043

[JEB240812C49] Smith, R. C. and Baker, K. S. (1981). Optical properties of the clearest natural waters (200–800 nm). *Appl. Opt.* 20, 177-184. 10.1364/AO.20.000177 20309088

[JEB240812C50] Smithers, S. P., Roberts, N. W. and How, M. J. (2019). Parallel processing of polarization and intensity information in fiddler crab vision. *Sci. Adv.* 5, eaax3572. 10.1126/sciadv.aax3572 31457103 PMC6703871

[JEB240812C51] Talbot, C. M. and Marshall, J. N. (2011). The retinal topography of three species of coleoid cephalopod: significance for perception of polarized light. *Phil. Trans. R. Soc. B* 366, 724-733. 10.1098/rstb.2010.0254 21282176 PMC3049017

[JEB240812C52] Temple, S. E., Pignatelli, V., Cook, T., How, M. J., Chiou, T.-H., Roberts, N. W. and Marshall, N. J. (2012). High-resolution polarisation vision in a cuttlefish. *Curr. Biol.* 22, R121-R122. 10.1016/j.cub.2012.01.010 22361145

[JEB240812C53] Temple, S. E., McGregor, J. E., Miles, C., Graham, L., Miller, J., Buck, J., Scott-Samuel, N. E. and Roberts, N. W. (2015). Perceiving polarization with the naked eye: characterization of human polarization sensitivity. *Proc. R. Soc. B* 282, 20150338. 10.1098/rspb.2015.0338 PMC4528539 26136441

[JEB240812C54] von Frisch, K. (1967). *The dance language and orientation of bees*. Cambridge, MA: Harvard University Press.

[JEB240812C55] Waterman, T. H., Fernández, H. R. and Goldsmith, T. H. (1969). Dichroism of photosensitive pigment in rhabdoms of the crayfish *Orconectes*. *J. Gen. Physiol.* 54, 415-432. 10.1085/jgp.54.3.415 5806598 PMC2225931

[JEB240812C56] Wehner, R. (1976). Polarized-light navigation by insects. *Sci. Am.* 235, 106-115. 10.1038/scientificamerican0776-106 935846

[JEB240812C57] York, T., Powell, S. B., Gao, S., Kahan, L., Charanya, T., Saha, D., Roberts, N. W., Cronin, T., Marshall, J., Achilefu, S. et al. (2014). Bioinspired polarization imaging sensors: from circuits and optics to signal processing algorithms and biomedical applications. *Proc. IEEE* 102, 1450-1469. 10.1109/JPROC.2014.2342537 PMC4629637 26538682

