# Supplementary material for: Thresholds of polarization vision in octopuses
Source: J Exp Biol. 2021 Apr 15;224(7):jeb240812. doi: 10.1242/jeb.240812 (PMC8077535; doi:10.1242/jeb.240812)
Supplement: Supplementary information [file jexbio-224-240812-s1.pdf]

Supplementary information

Movies 1 and 2. Supplementary videos of octopus responses to polarized stimuli

The animals were filmed from above and the stimulus was presented at what is the top of the screen when watching these videos.

Each movie shows examples of no response, very weak, weak, medium and strong responses. For each example the video will show 10 seconds of behaviour pre-stimulus presentation, followed by the response of the animal to the stimulus appearance and then disappearance 5 seconds later. The timing of the stimulus appearance and disappearance are demarcated with audible sounds (beeps). The movie then continues to show the response of the animal to the stimulus appearance again 3 times successively so that the observer can have a chance to observe the responses, which can sometimes be very subtle e.g. very weak responses.

Videos are also available to download from the University of Bristol data repository, data.bris, at https://doi.org/10.5523/bris.1r4kwj2eu0tnq1yj9b9pdeb5bg.

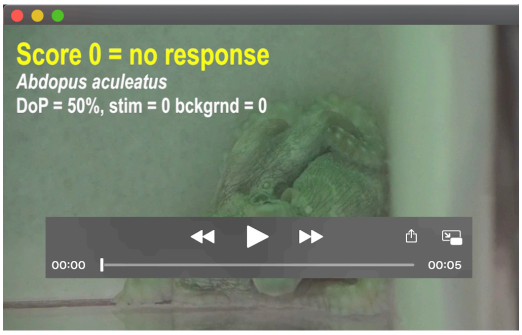

Movie 1

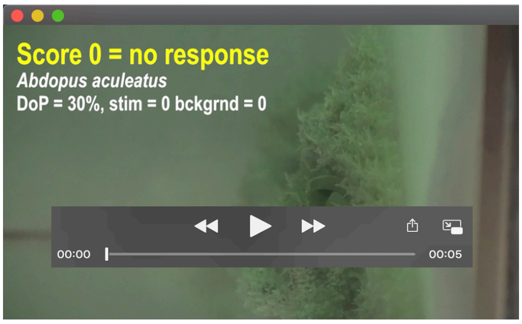

Movie 2

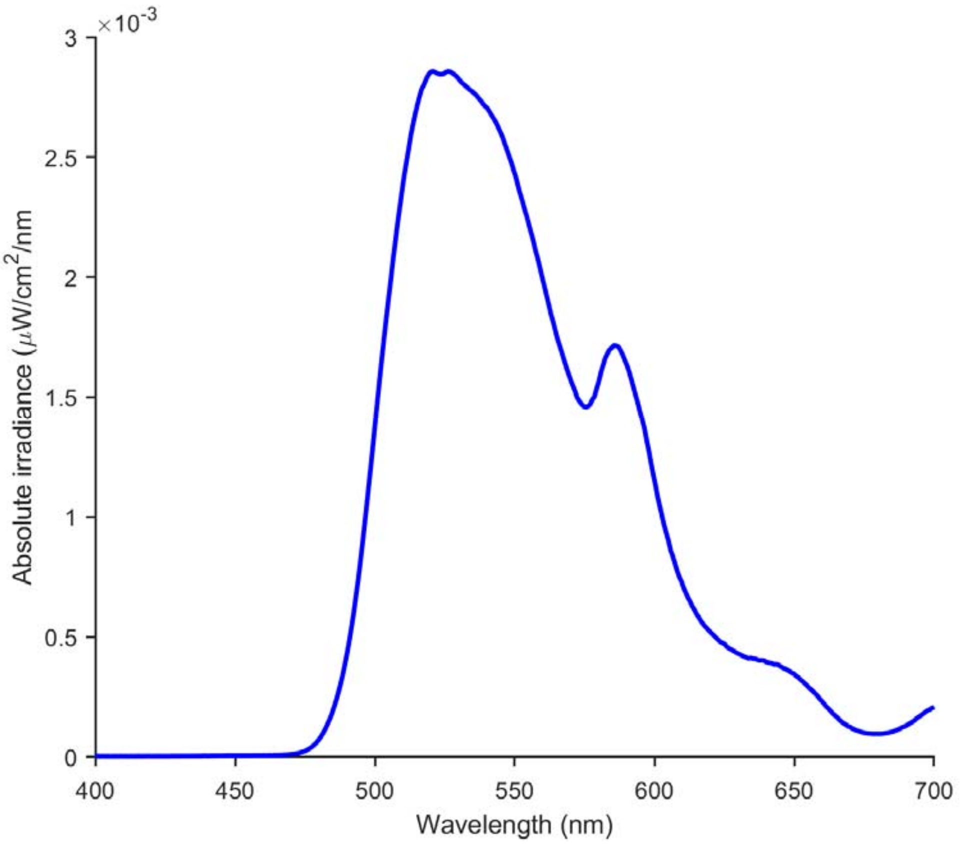

Figure S1. Absolute radiance measurement of light emitted from the modified LCD screen.

Journal of Experimental Biology • Supplementary information

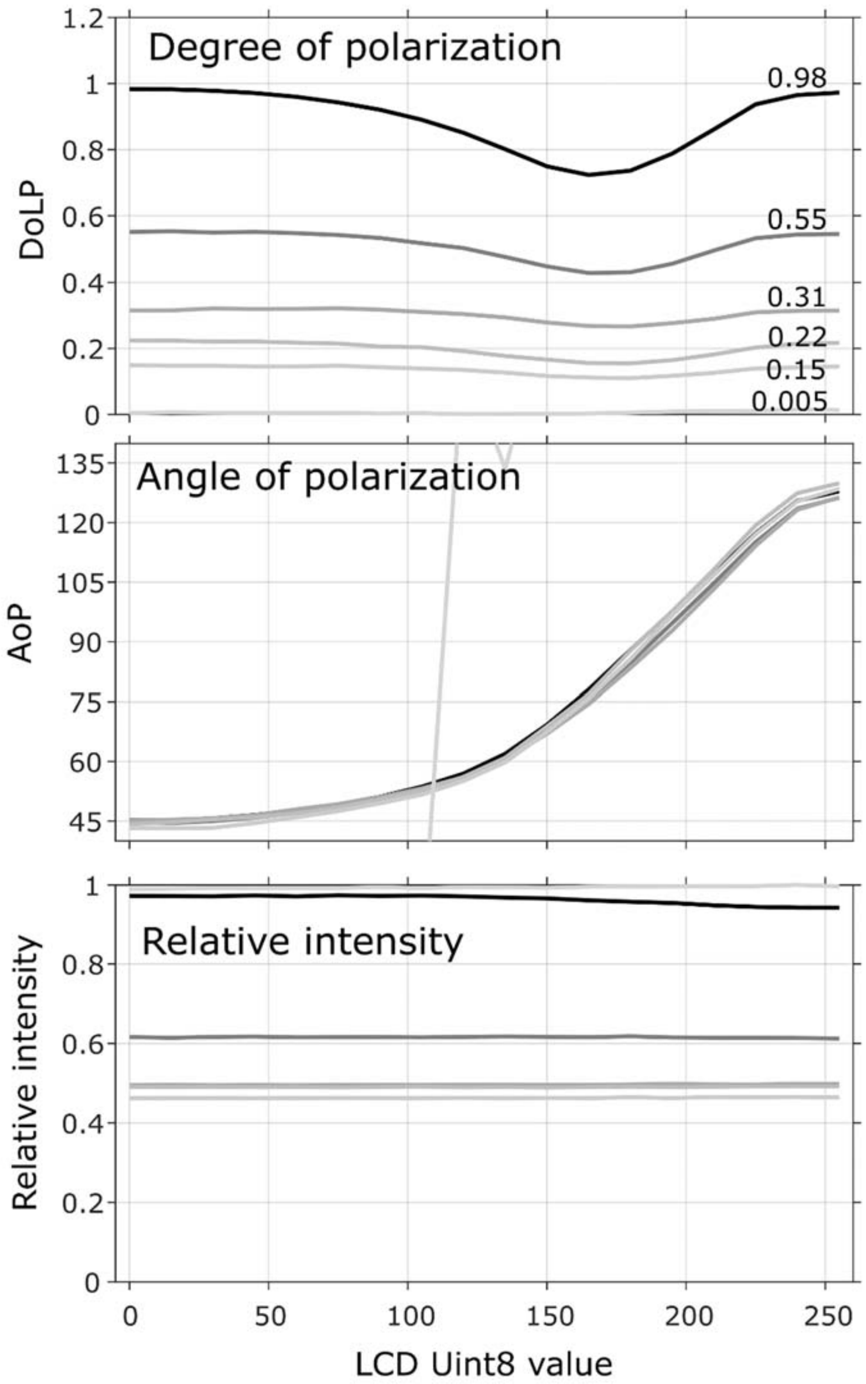

Figure S2. Expanded plot of the polarization and relative intensity characteristics of the stimulus system (including LCD monitor, DoLP filter and intervening neutral density filters (where applicable)). Measurements are presented for the six main DoLP levels used in the experiment, measured using a Glan-Thompson Fresnel-Rhomb assembly coupled to a spectrophotometer (USB2000, Ocean Optics). Note that the AoP estimates at the lowest DoLP setting are unreliable, resulting in the steep diagonal light grey line in the AoP graph.

Script 1. Matlab function for calculating polarization distance

```
function PD = pol2dist_2channel(varargin)

% function dist = pol2dist_2channel(obj_angle,obj_degree],...
%                           bg_angle,bg_degree,...
%                           [R1_ang,R2_ang],...
%                           PS,...
%                           receptorweight)
%
% Calculates polarization distance for two channel receptor
% inputs for a given polarization sensitivity (PS)
%
% Input format:
%
% obj_angle,obj_degree,...   object polarization angle and degree
%                           angle (radians)
%                           degree (0 to 1)
% bg_angle,bg_degree,...    background polarization angle and linear degree
%                           angle (radians)
%                           degree (0 to 1)
%
% receptors         vector of receptor angular sensitivities
%                   e.g: [0 pi/2] - orthogonal 2 channel array
%                        %
% PS        Polarization sensitivity of the receptors
%
% receptorweight   Weighting of receptor cells - default [1 1]
%
% Output format:
% structural variable PD with fields:
%
%   .max.R.Rs - Simulated receptor responses (for normalising)
%   .max.R.Rp - Simulated receptor potentials (for normalising)
%
%   .obj.R.Rs - Object receptor responses
%   .obj.R.Rp - Object receptor potentials
%   .obj.Rc - Receptor contrast (opponent signal between receptors)
%
%   .bg.R.Rs - Background receptor responses
%   .bg.R.Rp - Background receptor potentials
%   .bg.Rc - Background receptor contrast (opponent signal between
receptors)
%
%   .PD - calculated polarization distance
%   .Opp - raw opponent signal (difference between receptor contrasts Rc)
%   .P1o - normalised object receptor contrast (opponent signal)
%   .P1b - normalised background receptor contrast (opponent signal)
%
%Example: pol2dist_2channel(0,1,pi/2,1,[0 pi/2],10,[1 1])
%
% Written by Martin How, Nov 2012 - martin.j.how@gmail.com
% Modified by Martin How, Oct 2020 - m.how@bristol.ac.uk

rweight=[];

%Read in input variables
for aa = 1:size(varargin,2)

    obj_angle = varargin{1};
    obj_degree = varargin{2};
    bg_angle = varargin{3};
    bg_degree = varargin{4};
```

Journal of Experimental Biology • Supplementary information

```
    receptors = varargin{5};
    PS = varargin{6};
    if aa==7, rweight = varargin{7};end
end

%Assign default weight of [1 1] if unspecified
if size(rweight,2)<2, rweight = ones(size(receptors)); end

%%%%%%%%%%%%%%%%%%%%%%%%%%%%%%%%%%%%%%%%%%%%%%%%%%%%%%%%%%%%%%%%%%%%%%%%%%%
%Check input data format
if size(PS,1)~=1 || size(PS,2)~=1, error('PS must be a single value');end

%%%%%%%%%%%%%%%%%%%%%%%%%%%%%%%%%%%%%%%%%%%%%%%%%%%%%%%%%%%%%%%%%%%%%%%%%%%
%Run receptor absorbance calculation for object and background
for aa = 1:3
    if aa == 1, ang = receptors; deg = 1; PPS=10; end %Maximum for
normalising
    if aa == 2, ang = obj_angle; deg = obj_degree;PPS=PS; end
    if aa == 3, ang = bg_angle;  deg = bg_degree; PPS=PS;    end
    for rr = 1:size(receptors,2)
        rrr = receptors(rr);

        %Simulate receptor sensitivity (see subfunction at bottom of
        % script)
        R(rr).Rs = simulatereceptor(ang,deg,rrr,PPS,rweight(rr));


        %Receptor potential
        R(rr).Rp = log(R(rr).Rs);

    end

    if aa==1, PD.max.R = R; end
    if aa==2, PD.obj.R = R; end
    if aa==3, PD.bg.R = R;  end
end

%%%%%%%%%%%%%%%%%%%%%%%%%%%%%%%%%%%%%%%%%%%%%%%%%%%%%%%%%%%%%%%%%%%%%%%%%%%
%Two channel receptor contrast analysis
for aa = 1:2
    if aa == 1, R = PD.obj.R; end
    if aa == 2, R = PD.bg.R; end
    Rc = log(R(1).Rs./R(2).Rs);  %Receptor contrast

    if aa == 1, PD.obj.Rc = Rc; end
    if aa == 2, PD.bg.Rc = Rc;  end
end

PD.PD = abs(PD.obj.Rc-PD.bg.Rc)./(2*log(PS)); %Polarization distance
PD.Opp = PD.obj.Rc-PD.bg.Rc; %Opponent signal
PD.P1o = PD.obj.Rc./(2*log(PS))+0.5;
PD.P1b = PD.bg.Rc./(2*log(PS))+0.5;



%%%%%%%%%%%%%%%%%%%%%%%%%%%%%%%%%%%%%%%%%%%%%%%%%%%%%%%%%%%%%%%%%%%%%%%%%%%
function R_out = simulatereceptor(Q,deg,Qmax,PS,ww)

%Bernard and Wehner's receptor sensitivity function (1977 - Vision Research
% 17:1019-1028)
Rfun = @(x) 1+((deg.*(PS-1))./(PS+1)).*cos(2.*x-2.*Qmax);
R_out = feval(Rfun,Q).*ww;
```

Journal of Experimental Biology • Supplementary information
